# MicroRNA-17, 20a Regulates the Proangiogenic Function of Tumor-Associated Macrophages via Targeting Hypoxia-Inducible Factor 2α

**DOI:** 10.1371/journal.pone.0077890

**Published:** 2013-10-23

**Authors:** Zhenqun Xu, Lan Zhao, Ling-Yan Zhu, Min He, Limin Zheng, Yan Wu

**Affiliations:** 1 Key Laboratory of Gene Engineering of the Ministry of Education, State Key Laboratory of Biocontrol, School of Life Sciences, Sun Yat-sen University, Guangzhou, PR China; 2 Department of Clinical Laboratory, Guangdong Provincial Hospital of Chinese Medicine, Guangzhou, PR China; Queen Elizabeth Hospital, Hong Kong

## Abstract

Tumor-associated macrophages (TAMs) constitute a major component of the leukocyte infiltrate of most solid tumors, and they usually exhibit a proangiogenic phenotype which facilitates tumor growth in most circumstances. However, the precise mechanisms regulating the proangiogenic properties of TAMs remain largely unclear. In the present study, we found that the expression of hypoxia-inducible factor 2α (HIF-2α) was significantly up-regulated in macrophages from tumor tissues of several solid tumors. Macrophages exposed to tumor cell line derived-culture supernatants (TSN) also expressed high levels of HIF-2α in vitro, without a requirement for hypoxia. We identified miR-17 and miR-20a as the key regulators of HIF-2α expression in TAMs, and autocrine IL-6 played an important role in mediating the expression of miR-17, miR-20a, and thereafter HIF-2α in TAMs. Furthermore, the elevated HIF-2α in TAMs stimulated transcription of a set of proangiogenic genes such as VEGFA and PDGFB, which might in turn contribute to the angiogenic process within tumors. Our data provide evidence in support of the critical role of HIF-2α in the proangiogenic activity of TAMs and also reveal a novel mechanism by which miRNAs regulate TAM functions through modulation of HIF-2α expression under non-hypoxic conditions.

## Introduction

Hepatocellular carcinoma (HCC) is characterized by progressive development, high postsurgical recurrence, and extremely poor prognosis. The dismal outcome has been attributed to the highly vascular nature of HCC, which increases the propensity of tumor cells to spread and invade into neighboring or distant sites[1].

Although tumor cells were first thought to drive the cellular events underpinning tumor angiogenesis, considerable evidence has now emerged for the central role of macrophages in this process[2], [3]. In a previous study, we found that tumor environments can alter the normal development of macrophages that is intended to trigger transient early activation of monocytes in the peritumoral region, which in turn induces formation of suppressive macrophages in cancer nests[4]. Notably, the density of macrophages is selectively associated with vascular invasion and poor prognosis of HCC patients [5]–[11]. These results strongly indicate that, besides altering their differentiation and inflammatory status, signals derived from the tumor microenvironment might also drive macrophages to take on a proangiogenic phenotype, thus promoting the growth and spread of tumor cells, although the detailed mechanism remains largely unknown.

HIF-1α and HIF-2α are important hypoxia-induced factors that allow mammalian cells to adapt to changes in O_2_ availability[12]–[14]. While HIF-1α appears to be expressed ubiquitously, HIF-2α is expressed in a more tissue-restricted manner[15], [16]. It was reported that both HIF-1α and HIF-2α display pivotal activities in regulating cellular energy homeostasis, cell fate decisions, and oncogenesis[14], [17], [18]. However, HIF-2α in tumor-associated macrophages (TAMs) is specifically correlated with high-grade human tumors and poor prognosis[19], [20]. Despite the implications of HIF-2α indicated by these mentioned studies, the regulation and role of HIF-2α in TAMs is complex and incompletely described.

MicroRNAs (miRNAs) regulate a wide range of biological processes, including those linked to cancer and immunity[21], [22]. One of our recent studies showed that the tumor microenvironment can cause a sustained reduction in miR-155 in monocytes/macrophages, which in turn regulates the functional activities of these cells by increasing their expression of the transcription factor C/EBPβ[23]. These data implied an important role for miRNAs in determining the phenotype of macrophages in tumor tissues. Our present study identified another set of miRNAs – miR-17 and miR-20a– as key regulators of the transcription of proangiogenic genes in TAMs via directly targeting HIF-2α. The autocrine activity of IL-6 on TAMs played an important role in this process.

## Materials and Methods

### Patients and specimens

Tumor samples from 26 patients with pathologically confirmed HCC, 5 patients with lung cancer, and 4 patients with glioblastoma were obtained from the Cancer Center of Sun Yat-sen University. Of the HCC group, 11 were used for fresh CD14^+^ cell isolation, 9 were used for immunohistochemical analysis, and 6 were enrolled for immunofluorescence analysis. Samples from lung cancer and glioblastoma were used for immunohistochemical analysis. All tissues were obtained from patients undergoing resection. No local or systemic treatment had been conducted before the operations, and no other tumor or inflammatory disease was detected in these patients. Clinical stages were classified according to the International Union against Cancer and the clinical characteristics of these samples were summarized in Table S1-S3. All samples were anonymously coded in accordance with local ethical guidelines (as stipulated by the Declaration of Helsinki). Written informed consent was obtained from the patients and the protocol was approved by the Review Board of Sun Yat-sen University.

### Cell line and preparation of tumor culture supernatant

Human hepatocellular carcinoma cell line (HepG2) was obtained from the American Type Culture Collection; Human lung cancer cell line (95D), glioma cell line (U251) and normal liver cell line (L02) was obtained from the cell bank of the Committee on Type Culture Collection of Chinese Academic Science (CCTCC, Shanghai, China). All cells were tested for mycoplasma contamination using a single-step PCR method. The TSN or culture supernatant from normal liver cells were prepared as previously described[7].

### Isolation and culture of primary human monocytes/macrophages

Human peripheral blood mononuclear cells (PBMCs) were isolated from leukocyte-enriched buffy coats obtained from healthy donors, which have been tested to be negative for hepatitis B surface antigen, antibody to hepatitis C, antibody to HIV and serologic test for syphilis, by Ficoll density gradient centrifugation[4]. Monocytes were isolated from PBMCs by CD14 positive selection using magnetic-activated cell sorting (MACS) technology (Miltenyi Biotec, Bergisch Gladbach, Germany). The cells were cultured in complete medium consisting of DMEM supplemented with 10% human AB serum for 5–7 days to allow differentiation into macrophages. To generate TAM-like cells, monocytes were incubated with 15% cell-free supernatant of HepG2, or U251, or 95D cells, or with 20 ng/ml recombinant human IL-6 (R&D system, Minneapolis, MN, USA)[4], [24]. In some experiments, monocytes were incubated with 15% cell-free supernatant of L02 cells[7].

### Isolation of CD14^+^ cells from liver tissues

Fresh tumor- and non-tumor-infiltrating leukocytes were obtained as previously described[7]. In short, liver biopsy specimens (n = 7) were cut into small pieces and digested in RPMI 1640 supplemented with 0.05% collagenase IV (Sigma-Aldrich, St Louis, MO, USA), 0.002% DNase I (Roche Diagnostics, Mannheim, Germany), and 20% fetal calf serum (FCS; HyClone Laboratories, Logan, UT, USA) at 37°C for 20 min. Dissociated cells were filtered through a 150-µm mesh and separated by Ficoll density gradient, and CD14^+^ cells were purified by positive selection using anti-CD14 magnetic beads (Miltenyi Biotec).

### Immunohistochemical and immunofluorescence analyses

Paraffin-embedded samples from HCC, lung cancer or glioblastoma were cut into 5 µm sections, which were then processed for immunohistochemical analysis as previously described[25], [26]. Following incubation with anti-CD68 antibody (Ab; clone PG-M1, DakoCytomation, Glostrup, Denmark) or anti-HIF-2α Ab (Novus Biologicals, Littleton, CO, USA), the adjacent sections were stained using the Envision System (DakoCytomation, Glostrup, Denmark). For immunofluorescence analysis, frozen sections of human HCC tissues were double-stained with mouse anti-human CD68 Ab (DakoCytomation) and rabbit anti-human HIF-2α Ab (Novus Biologicals), and followed by Alexa Fluro 488-conjugated goat anti-mouse IgG and Alexa Fluro 555-conjuagted goat anti-rabbit IgG (Life Technologies Inc. Grand Island, NY, USA) [27]. Images were assessed with a scanning confocal microscope (TCS SP5, Leica, Wetzlar, Germany) and analyzed using LAS AF software (version 1.8.1).

### Immunoblotting

The cell lysates were extracted as previously described[28]. Equal amounts of cellular proteins were separated by 10% SDS-PAGE, immunoblotted with Abs against HIF-2α (Novus Biologicals), HIF-1α (clone 54, BD biosciences, San Jose, CA), IL-6R (Santa Cruz Biotechnology, Santa Cruz, CA, USA) and GAPDH (Abcam, Cambridge, UK), and visualized with an ECL kit (Thermo Fisher Scientific, Rockford, IL, USA).

### ELISA

Concentrations of IL-6 in the culture supernatants were determined using ELISA kits according to the manufacturer's instructions (eBioscience; San Diego, CA, USA).

### RNA extracts, real-time quantitative RT-PCR and gene expression analysis

Expression of mRNA and miRNA was determined by real-time quantitative reverse transcription-PCR (RT-PCR). Total RNA of cultured cells was isolated using TRIzol reagent (Life Technologies Inc.) and subsequently treated with RNase-free DNase I (Fermentas, Vilnius, Lithuania). Total RNA of tissue CD14^+^ cells was isolated using RNAqueous-Micro kit (Life Technologies Inc.). For mRNA analysis, total RNA (2 µg) was reverse-transcribed using MMLV reverse transcriptase (Fermentas), followed by SYBR Green real-time quantitative PCR. The specific primers used are listed in Table S4. Expression of mature miRNAs was detected with TaqMan miRNA assays according to the manufacturer's instruction (Life Technologies Inc.). All real-time quantitative PCR was performed on a Roche LightCycler480 (Roche Diagnostics) and data were analyzed using the LightCycler480 software (version 1.5.0). The level of target genes was normalized to the expression of reference genes (ACTB for mRNA and RNU6B for miRNAs), then the first control sample was used as a “calibrator” (target genes expression level set as 1), and all the other samples were compared to “calibrator”, which yield a 2^−ΔΔCt^ value.

### Oligonucleotides and transfection experiments

All RNA oligoribonucleotides were purchased from Genepharma (Shanghai, China). MiRNA duplexes corresponding to miR-17 and miR-20a were designed as described previously[29]. The small interfering RNAs (siRNAs) targeting human *EPAS1* (the HIF-2α coding gene) and *IL6R* (IL-6 receptor, IL-6R coding gene) transcripts were designated si-HIF-2α and si-IL-6R, respectively. The negative control RNA duplex (NC) for both miRNA mimic and siRNA was non-homologous to any human genome sequence. Anti-miR-17 and anti-miR-20a were 2′-O-methyl-modified oligoribonucleotides designed as inhibitors of miR-17 and miR-20a, respectively. Anti-miR-C was used as a negative control for anti-miRNAs in the antagonism experiment. The sequences of these oligonucleotides are listed in Table S4.

Transfection of RNA oligoribonucleotides was performed using HiPerFect (Qiagen, Hilden, Germany). Day-2 CD14^+^ cells were transfected with 200 nM oligonucleotides and incubated for 24 h. The culture medium was then replaced with fresh complete medium with or without 15% HepG2, 95D, or U251 TSN and incubated for 48 h. After that, the same transfection was repeated. The cells were processed for RNA or protein isolation on day 7.

### Vector construction and luciferase reporter assay

Three wild-type 3′UTR segments of human HIF-2α mRNA that contained the putative miR-17 and miR-20a binding sites were PCR-amplified (the sequences of primers are listed in Table S4) and inserted into the *Eco*RI/*Xba*I sites downstream of the stop codon of firefly luciferase in pGL3cm as previously described [30]. The resulting plasmids were denoted pGL3cm-HIF-2α-3′-UTR-1-WT, pGL3cm-HIF-2α-3′-UTR-2-WT and pGL3cm-HIF-2α-3′-UTR-3-WT, respectively. The plasmids pGL3cm-HIF-2α-3′-UTR-1-MUT, pGL3cm-HIF-2α-3′-UTR-2-MUT and pGL3cm-HIF-2α-3′-UTR-3-MUT, which carried mutated sequence in the complementary site for the seed region of miR-17 and miR-20a, were generated by site-specific mutagenesis based on pGL3cm- HIF-2α -3′-UTR-1-WT, pGL3cm- HIF-2α -3′-UTR-2-WT and pGL3cm- HIF-2α -3′-UTR-3-WT, respectively.

HepG2 cells seeded at 2×10^4^/well in 48-well plates were cotransfected with 10 ng of firefly luciferase reporter comprising wild-type or mutant 3′UTR of HIF-2α, 0.25 ng of pRL-CMV and 1 nM RNA duplex or 200 nm anti-miRNA oligoribonucleotides using Lipofectamine 2000 (Life Technologies Inc.). For the antagonism experiment, HepG2 cells seeded at 2×10^4^/well in 48-well plates were first transfected with 200 nM anti-miR-C or mixture of anti-miR-17 and anti-miR-20a. Twenty-four hours later, the RNA-transfected HepG2 cells were cotransfected with 10 ng of firefly luciferase reporter containing wild-type or mutant 3′-UTR of target gene and 0.25 ng of pRL-CMV. Cells were collected 48 h after the last transfection and analyzed using the Dual-Luciferase Reporter Assay System (Promega, Madison, WI, USA) as reported[30]-[32]. Luciferase activity was detected using an M200 microplate fluorescence reader (Tecan, Grodig, Austria). The pRL-CMV vector that provided the constitutive expression of *Renilla* luciferase was used as an internal control to correct for the differences in both transfection and harvest efficiencies. Transfections were carried out in duplicate and repeated at least three times in independent experiments.

### IL-6 blocking

Fresh blood monocytes were cultured for 2 h with 15% HepG2, 95D or U251 supernatant, and replaced with fresh complete medium. After 8 h, this conditioned medium was harvested and treated with 20 µg/mL IL-6 neutralizing Ab (clone 6708, R&D system) or a control IgG (clone 11711, R&D system) for 1 h, then used to stimulate differentiation of monocytes into macrophages[27].

### Statistical analysis

Results are expressed as means ± SEM or means ± SD. The statistical significance of differences between groups was determined by two-sided student's *t*-test and *P*<0.05 was considered statistically significant.

## Results

### Upregulation of HIF-2α expression in TAMs

**Figure 1:**
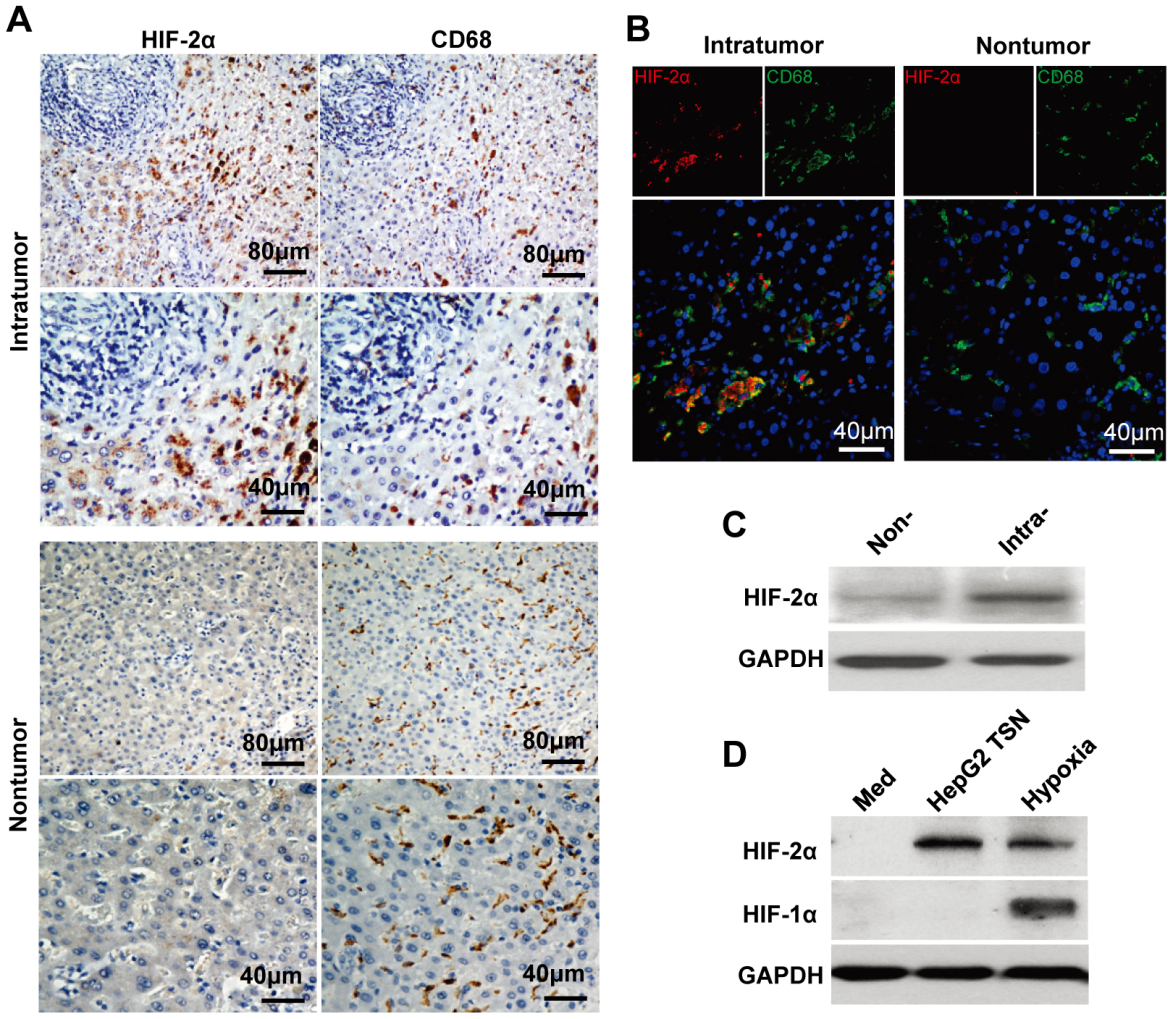
Upregulation of HIF-2α expression in TAMs. (A) Adjacent sections of paraffin-embedded HCC tissue (n = 9) were stained with an anti-HIF-2α or anti-CD68 antibody. (B) HIF-2α expression in macrophages from frozen sections of HCC tissue (n = 6) was analyzed by confocal microscopy. HIF-2α, red; CD68, green; DAPI, blue. (C) Level of HIF-2α protein in CD14^+^ cells isolated from non-tumoral (Non-) or intratumoral (Intra-) tissue of 4 HCC patients was analyzed by immunoblotting. (D) Healthy PBMC-derived monocytes were treated with medium alone (Med) for 7 days, or with HepG2 TSN under normoxic condition (HepG2 TSN) for 7 days, or with medium alone for 6 days and then exposed to 1% O_2_ for another 24 h (hypoxia). Expression of HIF-2α and HIF-1α was determined by Western blotting. This data shown are representative of four separate experiments.

To investigate the potential role of HIF-2α in carcinoma, we first examined its expression and distribution in serial sections of human HCC tumor samples stained for HIF-2α and CD68 (marker for monocytes/macrophages). As shown in Figure 1A and 1B, most macrophages showed marked expression of HIF-2α in the HCC cancer nest, while no HIF-2α was detected in adjacent normal tissue. Some tumor cells also exhibited expression of HIF-2α, but at a moderate level; and in some cases (4/9), HIF-2α was only expressed by macrophages in cancer nests, but not by tumor cells. HIF-2α expression in macrophages was further confirmed by western blot analysis showing that CD14^+^ cells isolated from intratumoral tissues exhibited higher expression of HIF-2α than that from non-tumoral tissues of HCC (Figure 1C). Moreover, most macrophages in lung cancer and glioblastoma were also positively stained with HIF-2α (Figure S1). These data suggest that tumor microenvironment may affect the HIF-2α expression in macrophages.

To test this assumption, monocytes of ∼98% purity were left untreated or cultured with supernatants from tumor (HepG2) or normal liver cell line cells (L02) for 7 days, under normoxic or hypoxic conditions. Interestingly, while hypoxia induced both HIF-1α and HIF-2α expression in macrophages, tumor supernatants (TSN) under normoxic condition induced only the expression of HIF-2α. Normal liver cell line supernatants showed no effect on macrophages expression of either HIF-1α or HIF-2α (Figure 1D and S2). These data indicate that compared with HIF-1α, HIF-2α might be first induced in monocytes/macrophages by soluble factors derived from tumor microenvironment, even before these cells encounter hypoxia. Consistent with the above data, supernatants from lung cancer (95D) and glioblastoma (U251) tumor cell line were also capable of increasing the expression of HIF-2α in monocyte-derived macrophages (Figure S2).

### MiR-17 and miR-20a regulate HIF-2α expression at post-transcriptional level in TAMs

**Figure 2:**
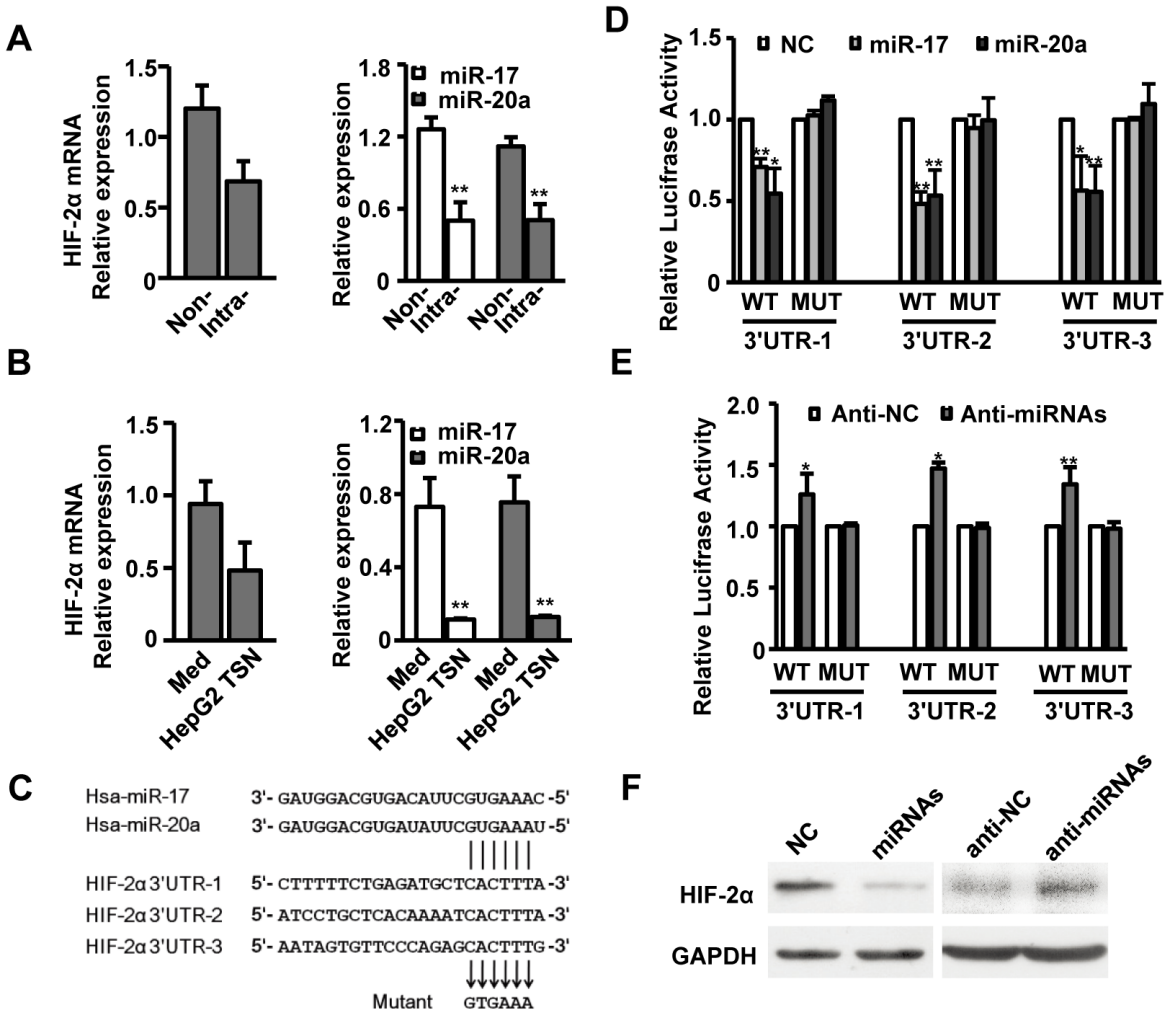
MiR-17 and miR-20a regulate HIF-2α expression at the post-transcriptional level in TAMs. (A, B) CD14^+^ cells were isolated from non-tumoral (Non-) or intratumoral (Intra-) tissues of seven HCC patients (A). Healthy PBMC-derived CD14^+^ cells were treated with medium alone or HepG2 TSN for 7 days (B). Levels of HIF-2α mRNA, miR-17 and miR-20a in these cells were analyzed by qPCR. Data were presented as relative level and normalized to ACTB for mRNA expression or RNU6B for miRNAs expression. (C) Pairing of miR-17 and miR-20a with three HIF-2α 3′UTR regions (nucleotides 170–176, 816–822 and 1500–1506 of human HIF-2α 3′UTR), and mutations introduced into the luciferase reporter construct are shown. (D, E) Luciferase activity of HepG2 cells transfected with the indicated reporters was analyzed. pRL-CMV expressing *Renilla* luciferase was cotransfected as an internal control and the luciferase activity of each sample was normalized to this. The luciferase activity of the NC or anti-miR-C transfectant was assigned an arbitrary value of 1. (F) Healthy PBMC-derived CD14^+^ cells were treated with medium alone and then transfected with NC or anti-miR-17 and anti-miR-20a mixture (anti-miRNAs), or with HepG2 TSN and then transfected with NC or miR-17 and miR-20a mixture (miRNAs). HIF-2α expression was analyzed by Western blotting. Values represent the mean ± SEM for A and mean ± SD for the others. All data shown are representative of at least four separate experiments. *P<0.05, **P<0.01 compared with the indicated groups.

To further elucidate mechanisms regulating HIF-2α expression in macrophages, we analyzed HIF-2α mRNA levels in macrophages isolated from non-tumor and intratumoral areas of HCC tissues. While HIF-2α expression markedly increased in intratumoral macrophages (Figure 1), its mRNA level was similar to that detected in non-tumor-infiltrating macrophages (Figure 2A), which indicates that HIF-2α is post-transcriptionally regulated in TAMs. By target prediction programs TargetScan and PicTar, a set of potential miRNAs that may target HIF-2α were identified, including miR-17 and miR-20a, which are located in the same miRNA cluster and share identical seed sequences. As expected, levels of both miR-17 and miR-20a were significantly decreased in intratumoral macrophages compared to that detected in non-tumor-infiltrating macrophages (n = 7, Figure 2A). The downregulation of miR-17 and miR-20a was further confirmed by in vitro culture models showing that with similar HIF-2α mRNA levels, miR-17 and miR-20a were markedly decreased in macrophages treated with HepG2 TSN (Figure 2B). Consistent with these results, the expression of miR-17 and miR-20a was also down-regulated in macrophages treated with 95D or U251 TSN, while the mRNA level of HIF-2α was not significantly different from the control group (Figure S3A and B). These data indicate that miR-17 and miR-20a mediate post-transcriptional suppression of HIF-2α expression in macrophages.

To confirm this hypothesis, we employed dual-luciferase reporter system and cloned the human HIF-2α 3′UTR fragment, including three wild-type and their respective mutant putative binding sites for miR-17 and miR-20a, downstream of the firefly luciferase gene (Figure 2C). As shown in Figures 2D and 2E, transfection with miR-17 or miR-20a significantly suppressed the luciferase activity of all three wild-type reporters, while inhibition of endogenous miR-17 and miR-20a with a mixture of anti-miR-17 and anti-miR-20a led to increased luciferase activity of these wild-type reporters. Mutant reporters showed no such effects. These data imply that miR-17 and miR-20a directly regulate HIF-2α expression through the three putative binding sites at the HIF-2α 3′UTR.

In light of these results, we further explored the endogenous effects of miR-17 and miR-20a on HIF-2α expression in HepG2 TSN-treated macrophages. As shown in Figure 2F and S3C, transfection of the miR-17 and miR-20a mixture markedly decreased HIF-2α expression in macrophages treated with TSN. In contrast, transfection of anti-miR-17 and anti-miR-20a mixture showed an opposing effect by significantly increasing the expression of HIF-2α. These results confirm that miR-17 and miR-20a regulate HIF-2α expression by directly targeting its 3′UTR in TAMs.

### Role of IL-6 in downregulation of miR-17, miR-20a and upregulation of HIF-2α expression in TAMs

**Figure 3:**
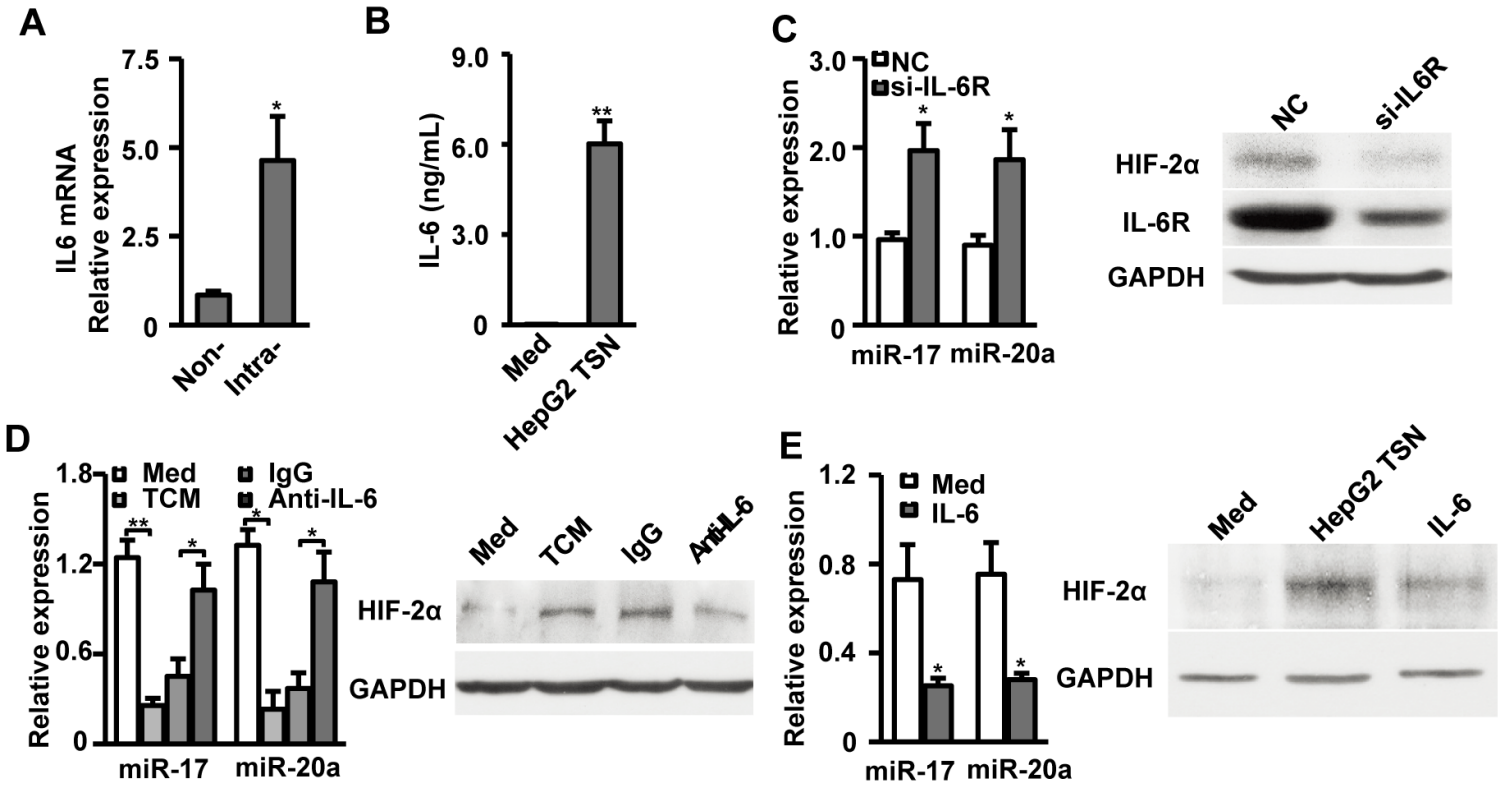
Role of IL-6 in downregulation of miR-17 and miR-20a and upregulation of HIF-2α expression in TAMs. (A) Level of IL-6 mRNA in CD14^+^ cells isolated from non-tumoral (Non-) or intratumoral (Intra-) tissue of seven HCC patients was analyzed by qPCR. Results are presented as fold-changes. (B) Healthy PBMC-derived CD14^+^ cells were treated with medium alone or HepG2 TSN for 24h. IL-6 concentrations in the culture supernatants were determined by ELISA. (C, D, E) Healthy PBMC-derived CD14^+^ cells were treated HepG2 TSN and transfected with NC or si-IL-6R (C). Healthy PBMC-derived CD14^+^ cells were treated with medium, TCM, TCM with anti-IL-6, or TCM with IgG control for 7 days (D). Healthy PBMC-derived CD14^+^ cells were treated with medium alone or recombinant IL-6 for 7 days (E). MiR-17 and miR-20a in these cells were analyzed by qPCR. Expression of HIF-2α was determined by Western blotting. qPCR data were presented as fold-changes and normalized to ACTB for mRNA expression or RNU6B for miRNAs expression. Values represent the mean ± SEM for A and mean ± SD for the others. All data shown are representative of at least four separate experiments. *P<0.05, **P<0.01 compared with the indicated groups.

IL-6 has been implicated in regulating the expression of HIF-1 in some tumor cells[33]. Both intratumor-infiltrating macrophages and in vitro HepG2-TSN-exposed macrophages expressed large amounts of IL-6 (Figure 3A and 3B) while supernatants from HepG2 expressed low levels of IL-6 itself (data not shown). Based on these data, we hypothesize that tumor-induced autocrine IL-6 production might play a role in regulating miR-17, miR-20a, and thereafter HIF-2α expression in TAMs. We performed three sets of experiments to test this assumption:

First, monocytes were transfected with si-IL6R prior to exposure to HepG2 TSN. The results showed that knockdown of IL-6R in macrophages block the downregulation of miR-17 and miR-20a, and decreased the level of HIF-2α expression compared to NC-transfected controls (Figure 3C).

Next, conditioned medium from HepG2 TSN-exposed macrophages (TCM) was used to stimulate monocytes instead of HepG2 TSN. The results showed that TCM exerted similar effects on miR-17, miR-20a, and HIF-2α expression in macrophages as HepG2 TSN. Blockade of IL-6 with an IL-6-neutralizing antibody efficiently restored levels of miR-17, miR-20a (approximate 2.28-fold upregulated and 2.92-fold upregulated respectively), and abrogated induction of HIF-2α expression in macrophages compared to the IgG control group (Figures 3D and S4).

In the third set of experiments, recombinant human IL-6 was used to stimulate monocytes. As shown in Figure 3E, IL-6 induced a decrease in miR-17 and miR-20a, thus leading to increased HIF-2α expression in macrophages. Together, these data clearly show that autocrine IL-6 is involved in the downregulation of miR-17 and miR-20a and the upregulation of HIF-2α expression in TAMs.

### HIF-2α modulates proangiogenic gene expression in TAMs

**Figure 4:**
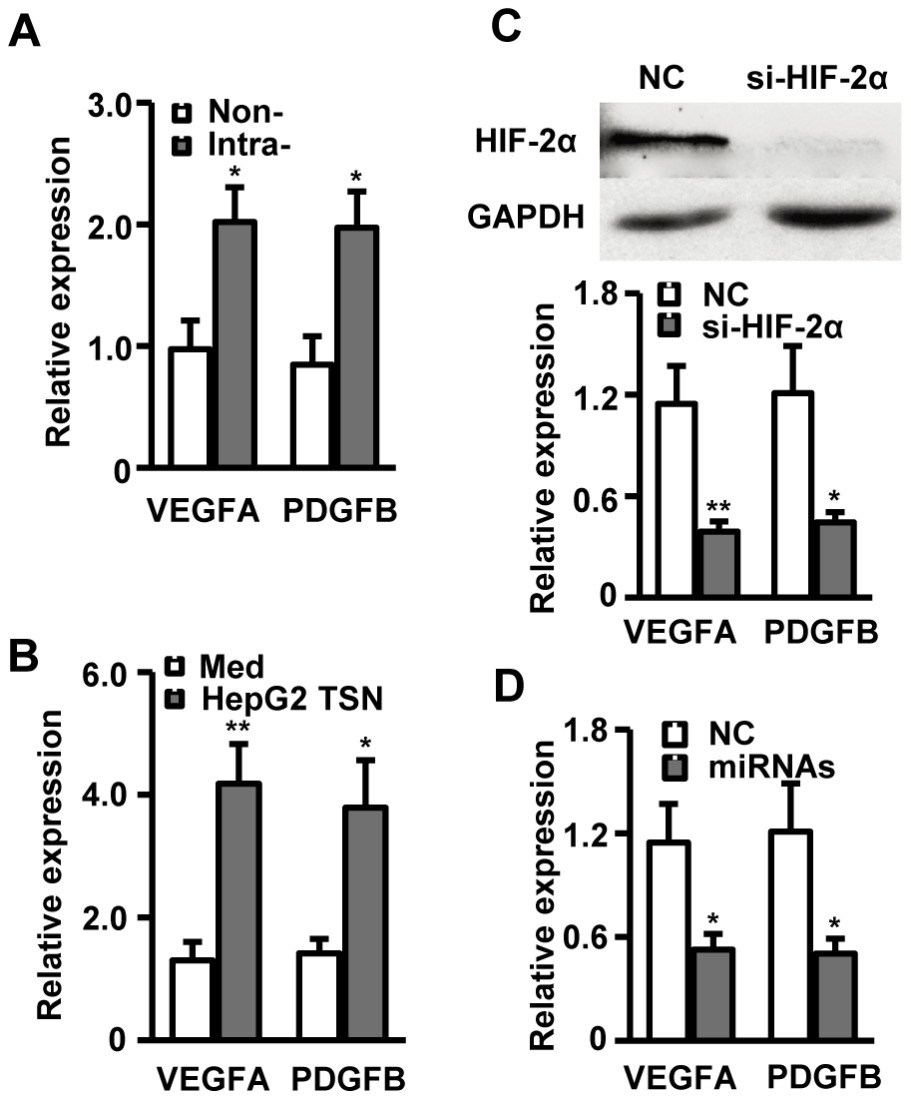
HIF-2α modulates proangiogenic gene expression in TAMs. (A) CD14^+^ cells were isolated from non-tumoral (Non-) or intratumoral (Intra-) tissue of seven HCC patients. (B) Healthy PBMC-derived CD14^+^ cells were treated with medium alone or HepG2 TSN for 7 days. (C, D) Healthy PBMC-derived CD14^+^ cells were treated with HepG2 TSN and transfected with NC, si-HIF-2α (C), or miR-17 and miR-20a mixture (D). Levels of VEGFA and PDGFB mRNA expression in these cells were analyzed by qPCR. Upper panel of C shows the knockdown efficiency of si-HIF-2α. All results are normalized to ACTB and presented as fold-changes. All data shown are representative of at least four separate experiments. *P<0.05, **P<0.01 compared with the indicated groups.

The results described above showed that macrophages significantly upregulated HIF-2α expression following exposure to HepG2 TSN without a requirement for hypoxia. Therefore, we went on to investigate whether HIF-2α in TAMs still possessed proangiogenic capability via transcription of proangiogenic genes as it did under hypoxic conditions. As shown in Figure 4A, macrophages isolated from HCC tumor tissue expressed significantly higher levels of VEGFA and PDGFB mRNA compared to those detected in non-tumor-infiltrating macrophages (approximate 2.07-fold upregulated, *P* = 0.015 and approximate 2.33-fold upregulated, *P* = 0.011, respectively, n = 7). The increase in VEGFA and PDGFB was further confirmed in cultured macrophages exposed to HepG2 TSN in vitro (approximate 3.21-fold upregulated, *P* = 0.007 and approximate 2.68-fold upregulated, *P* = 0.026, respectively, Figure 4B). Transfection of si-HIF-2α abrogated TSN-induced upregulation of VEGFA and PDGFB expression in macrophages (Figure 4C), and transfection of a mixture of miR-17 and miR-20a showed similar effects to that of si-HIF-2α (Figure 4D). These data strongly indicate that HIF-2α is an active transcriptional effecter that could promote transcription of proangiogenic genes in TAMs under non-hypoxic conditions.

Consistent with these results, macrophages treated with 95D or U251 TSN also produced large amount of IL-6, and blockage of IL-6 with an IL-6-neutralizing antibody efficiently restored levels of miR-17 and miR-20a expression in macrophages exposed to 95D or U251 TCM (Figure S5). Moreover, macrophages treated with 95D or U251 TSN significantly up-regulated the expression of VEGFA and PDGFB, and transfection of si-HIF-2α or a mixture of miR-17 and miR-20a could also markedly down-regulate the expression of these proangiogenic genes in TAMs (Figure S6).

## Discussion

**Figure 5:**
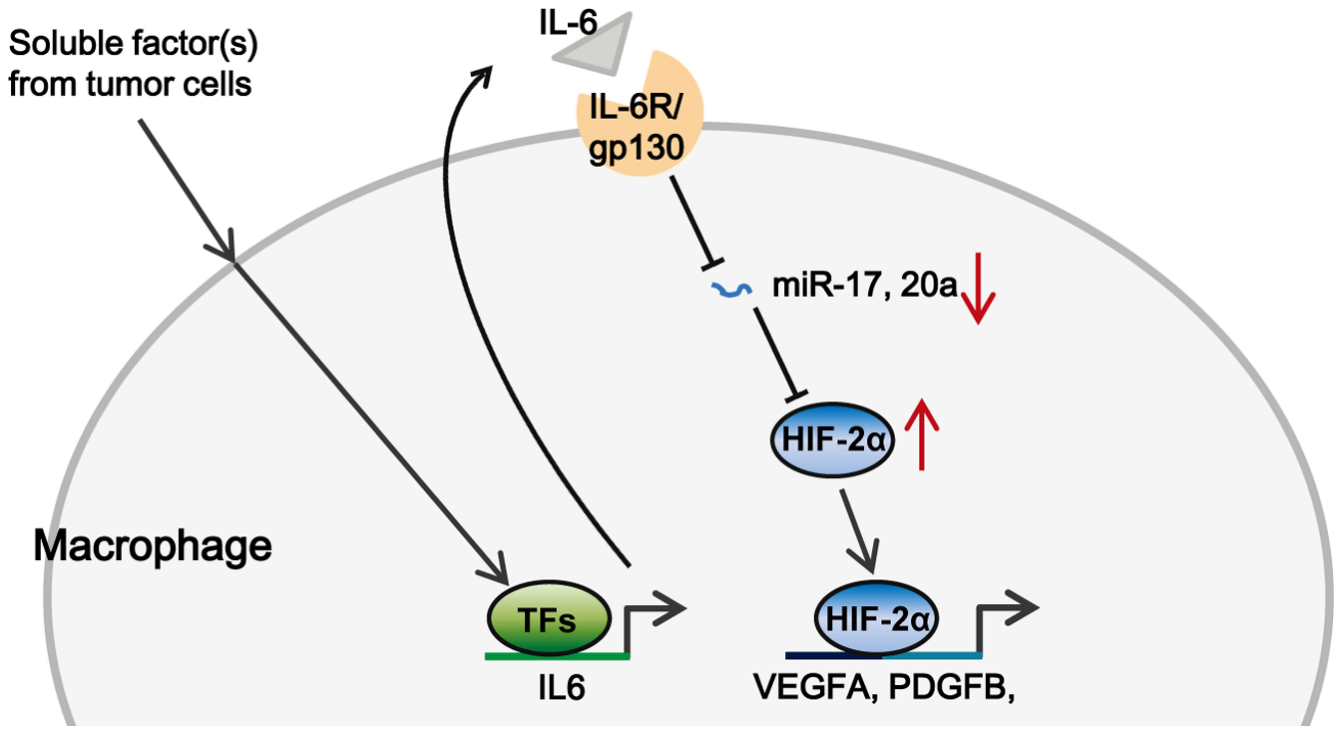
Model of the novel mechanism regulating HIF-2α expression under non-hypoxic condition within tumor microenvironment. Soluble factor(s) derived from tumor cells induced the secretion of IL-6 by stimulating its transcription. Autocrine IL-6 triggered the down-regulation of miR-17 and miR-20a, which in turn released the suppression of HIF-2α and led to its accumulation under non-hypoxic condition. HIF-2α further promoted the transcription of proangiogenic genes such as VEGFA and PDGFB and contributed to tumor angiogenesis.

HIF-2α regulates both the migration and proinflammatory cytokine/chemokine production of TAMs, and its overexpression is usually correlated with high-grade human tumors and poor prognosis[16], [19], [20]. However, the precise mechanisms regulating HIF-2α expression in TAMs remains largely unclear. The present study provides evidence that miR-17 and miR-20a directly regulates HIF-2α expression in TAMs by targeting the 3′UTR of HIF-2α mRNA. The downregulation of miR-17 and miR-20a, and upregulation of HIF-2α in TAMs cultured in vitro were independent of hypoxia, and HIF-2α in these macrophages was still capable of promoting transcription of a set of proangiogenic genes, indicating its regulatory role in the proangiogenic functions of TAMs within a tumor (Figure 5).

MiRNAs have been found to modulate most cellular processes in diverse cell types and their dysregulation is associated with different diseases[34]-[38]. As for macrophages in a tumor microenvironment, our previous study showed that miR-155 regulated inflammatory cytokine production, including IL-6, in TSN-exposed monocytes via targeting C/EBPβ [23]. Other investigators have reported that miRNA-17, miR-20a and miR-106a increase the expression of the M-CSF receptor (M-CSFR) in macrophages by targeting RUNX1[39], which could promote the differentiation of macrophages into M2/TAM-like cells[24]. Our current study complements these findings by showing that miRNA-17 and miR-20a regulated HIF-2α expression in TAMs, and therefore may be involved in the regulation of the proangiogenic functions of TAMs within tumors. These data suggest the pivotal and diverse roles of miRNAs in regulating TAMs functions, and selective modulation of one or a combination of different miRNAs in macrophages might provide more a specific strategy for anticancer therapies.

Both HIF-1α and HIF-2α accumulate in hypoxic primary human macrophages, and were reported to have non-overlapping roles[14], [16]. While HIF-1α mediates macrophage-dependent inflammatory and antibacterial activities, HIF-2α is involved in the regulation of TAM migration and proinflammatory cytokine/chemokine production[16]. In the current study, in the absence of hypoxia, macrophages expressed HIF-2α, but not HIF-1α after exposure to TSN, and HIF-2α in these macrophages promoted the transcription of a set of proangiogenic genes, which in turn might regulate the proangiogenic functions of TAMs. These data indicate that TAMs promote angiogenesis earlier than previously thought, perhaps even before hypoxia emerges. Consistent with our results, other investigators have reported that, compared to HIF-1α, HIF-2α is more stable under high oxygen tension in non-malignant/malignant cells[40]–[42], and this protein escapes degradation under near-normoxic conditions in breast cancer[43].

Both intratumor-infiltrating macrophages and in vitro HepG2-TSN-exposed macrophages produced large amounts of IL-6. The present study provides evidence in support of the important role played by autocrine IL-6 in regulating miR-17 and miR-20a, and thereafter, HIF-2α expression in TAMs. First, conditioned medium from HepG2-TSN-exposed macrophages (TCM) exerted similar effects on miR-17, miR-20a, and HIF-2α expression in macrophages as did HepG2 TSN. Furthermore, blockade of IL-6 with an IL-6-neutralizing antibody efficiently restored levels of miR-17 and miR-20a, and reduced the HIF-2α expression. Second, knockdown of IL-6R in macrophages exhibited similar effects to that observed in the presence of an IL-6-neutralizing antibody. Third, recombinant human IL-6 decreased levels of miR-17 and miR-20a, and thus increased HIF-2α expression in macrophages. These data clearly suggest a role for autocrine IL-6 in mediating HIF-2α expression via downregulation of miR-17 and miR-20a in TAMs. Interestingly, there is substantial evidence that HIF-2α induces the production of IL-6 in macrophages[16]. Therefore, the expression of HIF-2α might be further increased in TAMs due to the availability of increased IL-6 as a result of a positive feedback regulatory loop.

It should be noted that the network regulating the proangiogenic function of TAMs is much more complex than we have already explored. For example, more than a dozen target genes have been validated for miR-17 and miR-20a, including HIF-1α, IL-8, and VEGFA[44]-[47]. Moreover, HIF-2α is capable of promoting the transcription of genes that are relevant/irrelevant to angiogenesis as well as VEGFA and PDGFB. Therefore, characterization of the intricate mechanisms underlying the proangiogenic function of TAMs may provide new avenues for development of novel immune-based therapies in human cancer.

Although mainly focused on hepatocellular carcinoma, the current study provided important evidence that similar regulatory loop might also apply to TAMs from other solid tumors like lung cancer and glioblastoma. It has been reported by other groups that IL-6, VEGFA and PDGFDB were significantly up-regulated in macrophages isolated from tissues of human lung cancer and glioblastoma[48]–[51]. These data were, in part, consistent with our current conclusions.

TAMs are derived from circulating monocytes, and, through continual interaction with and promotion of differentiation by tumor microenvironmental signals, these cells acquire special phenotypic characteristics with diverse functions[3], [52]–[54]. Our results give important new insights into the regulation of the proangiogenic functions of TAMs in tumors. Autocrine IL-6 triggered the downregulation of miR-17 and miR-20a, which in turn led to the accumulation of HIF-2α in TAMs. HIF-2α in these cells transcribed a set of proangiogenic genes which might contribute to the process of angiogenesis within a tumor. Therefore, it is possible that studies of the mechanisms that selectively modulate levels of one or a set of key miRNAs in TAMs will provide a novel strategy for anticancer therapy.

## Supporting Information

Figure S1 **Macrophages expression of HIF-2α in lung cancer and glioblastoma.** Adjacent sections of paraffin-embedded tissue from lung cancer (n = 5) (A) or glioblastoma (n = 4) (B) were stained with an anti-HIF-2α or anti-CD68 antibody.
(TIF)

Figure S2 **Effects of tumor supernatant on HIF-2α expression in macrophages.** Healthy PBMC derived monocytes were left untreated or treated with indicated tumor supernatant for 7 days. HIF-2α expression was determined by Western blotting. Data shown are representative of four separate experiments.
(TIF)

Figure S3 **Regulation of HIF-2α expression by MiR-17 and miR-20a in macrophages treated with 95D or U251 TSN.** (A, B) Healthy PBMC derived monocytes were left untreated or treated with indicated tumor supernatant for 7 days. Levels of HIF-2α mRNA (A), miR-17 and miR-20a (B) were determined by qPCR. (C) Healthy PBMC derived monocytes were treated with indicated tumor supernatant and then transfected with NC or miR-17 and miR-20a mixture (miRNAs). HIF-2α expression was analyzed by Western blotting. Data shown are representative of four separate experiments. Values represent the mean ± SEM for A and B. *P<0.05, **P<0.01 compared with the indicated groups.
(TIF)

Figure S4 **Blocking effect of anti-IL-6 antibody.** TCM were untreated, treated with anti-IL-6, or an isotype (IgG) control for 1 h. IL-6 concentrations were determined by ELISA. Values represent the mean ± SEM of four separate experiments. ***P<.001 compared with the indicated groups.
(TIF)

Figure S5 **Role of IL-6 in downregulation of miR17 and miR20a in TAMs.** (A) Healthy PBMC derived monocytes were treated with medium alone (MO), 95D TSN (95D TSN-MO), or U251 TSN (U251 TSN-MO) for 24 hours. IL-6 concentrations in the culture supernatants were determined by ELISA. (B) Healthy PBMC-derived monocytes were treated with medium, conditioned medium from 95D or U251 TSN-exposed macrophages (TCM), 95D or U251 TCM with anti-IL-6, or 95D or U251 TCM with IgG control for 7 days. MiR-17 and miR-20a in these cells were analyzed by qPCR. Values represent the mean ± SEM of four separate experiments. *P<0.05, **P<0.01 compared with the indicated groups.
(TIF)

Figure S6 **Regulation of VEGFA, PDGFB expression by HIF-2α, miR17 and miR20a in macrophages treated with 95D or U251 TSN.** (A) Healthy PBMC derived monocytes were treated with medium alone, 95D TSN, or U251 TSN for 7 days. (B, C) Healthy PBMC derived monocytes were treated with 95D or U251 TSN and transfected with NC, si-HIF-2α (B), or miR-17 and miR-20a mixture (miRNAs) (C). Levels of VEGFA and PDGFB mRNA expression in these cells were analyzed by qPCR. Values represent the mean ± SEM of four separate experiments. *P<0.05, **P<0.01 compared with the indicated groups.
(TIF)

Table S1 **Clinical characteristics of the 26 HCC patients.**
(DOCX)

Table S2 **Clinical characteristics of the 5 lung cancer patients.**
(DOCX)

Table S3 **Clinical characteristics of the 4 glioblastoma patients.**
(DOCX)

Table S4 **Sequences of RNA and DNA Oligonucleotides.**
(DOCX)
