# Supplementary figures and images for: MicroRNA-17, 20a Regulates the Proangiogenic Function of Tumor-Associated Macrophages via Targeting Hypoxia-Inducible Factor 2α
Source: PLoS One. 2013 Oct 23;8(10):e77890. doi: 10.1371/journal.pone.0077890 (PMC3806827; doi:10.1371/journal.pone.0077890)

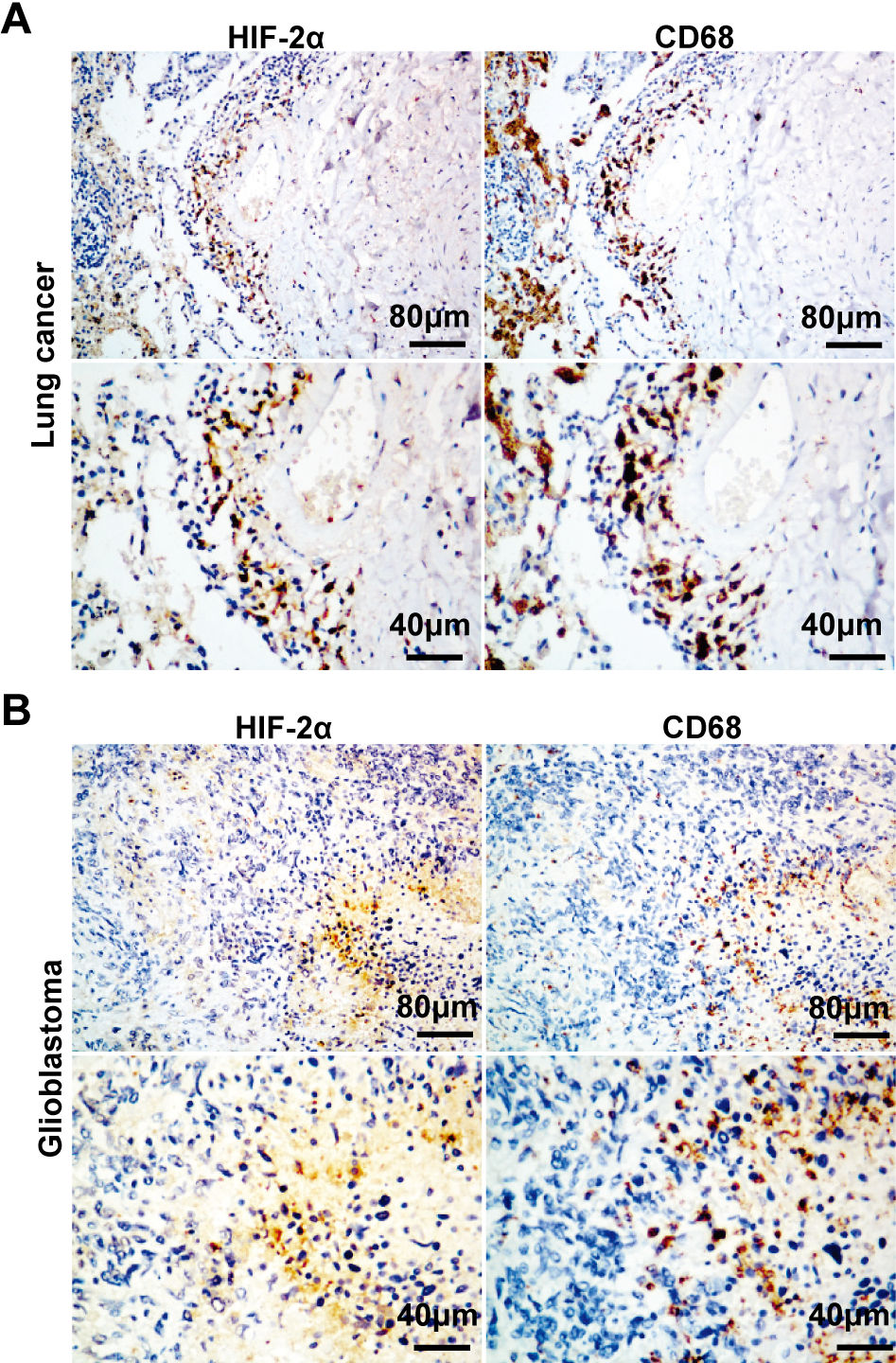

Supplement: Figure S1 — Macrophages expression of HIF-2α in lung cancer and glioblastoma. Adjacent sections of paraffin-embedded tissue from lung cancer (n = 5) (A) or glioblastoma (n = 4) (B) were stained with an anti-HIF-2α or anti-CD68 antibody. (TIF) [file pone.0077890.s001.tif]

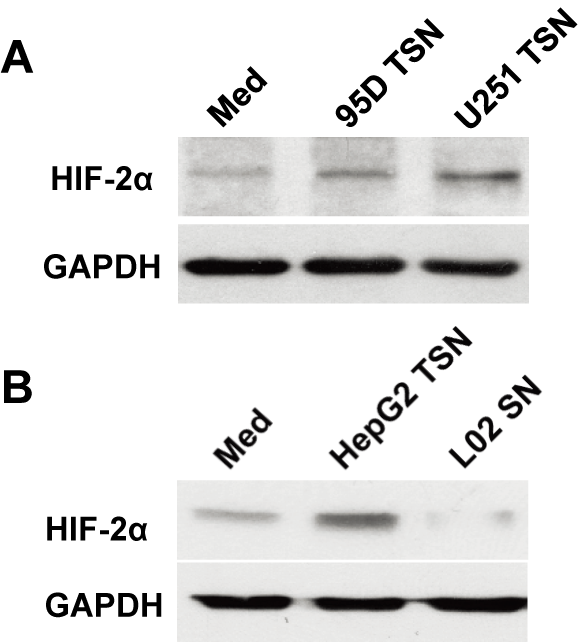

Supplement: Figure S2 — Effects of tumor supernatant on HIF-2α expression in macrophages. Healthy PBMC derived monocytes were left untreated or treated with indicated tumor supernatant for 7 days. HIF-2α expression was determined by Western blotting. Data shown are representative of four separate experiments. (TIF) [file pone.0077890.s002.tif]

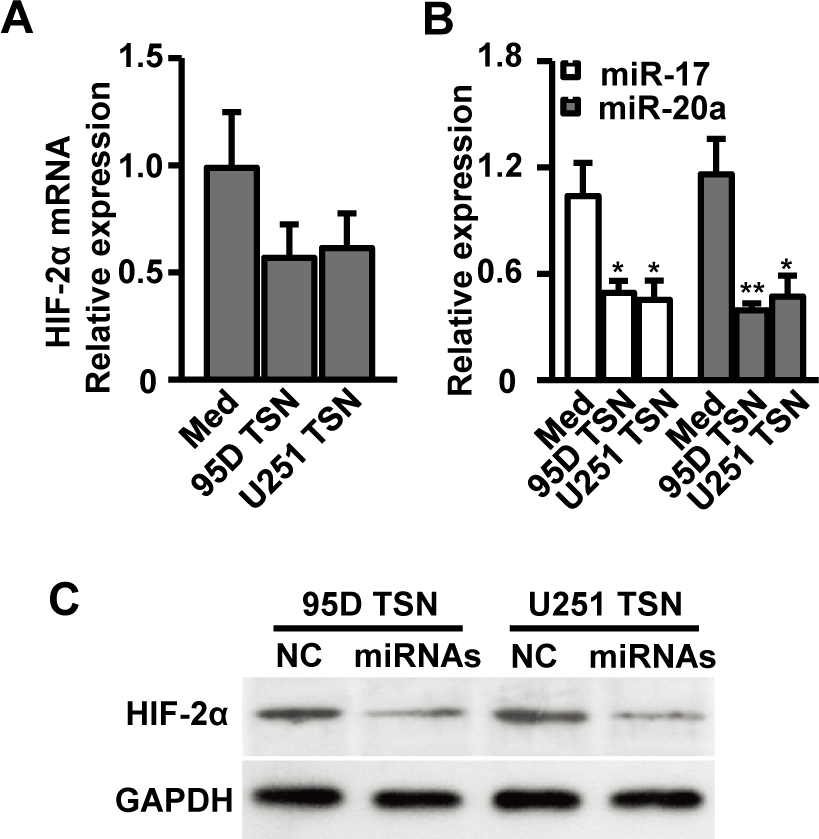

Supplement: Figure S3 — Regulation of HIF-2α expression by MiR-17 and miR-20a in macrophages treated with 95D or U251 TSN. (A, B) Healthy PBMC derived monocytes were left untreated or treated with indicated tumor supernatant for 7 days. Levels of HIF-2α mRNA (A), miR-17 and miR-20a (B) were determined by qPCR. (C) Healthy PBMC derived monocytes were treated with indicated tumor supernatant and then transfected with NC or miR-17 and miR-20a mixture (miRNAs). HIF-2α expression was analyzed by Western blotting. Data shown are representative of four separate experiments. Values represent the mean ± SEM for A and B. *P<0.05, **P<0.01 compared with the indicated groups. (TIF) [file pone.0077890.s003.tif]

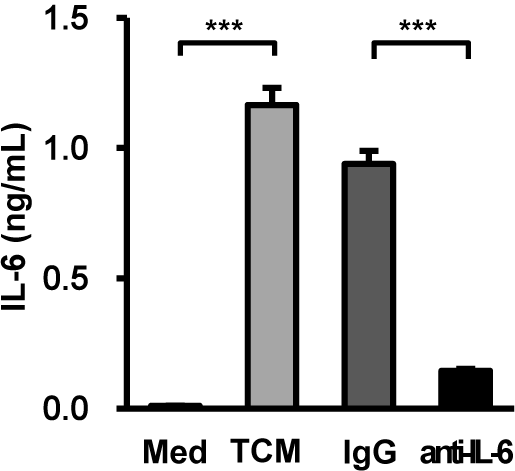

Supplement: Figure S4 — Blocking effect of anti-IL-6 antibody. TCM were untreated, treated with anti-IL-6, or an isotype (IgG) control for 1 h. IL-6 concentrations were determined by ELISA. Values represent the mean ± SEM of four separate experiments. ***P<.001 compared with the indicated groups. (TIF) [file pone.0077890.s004.tif]

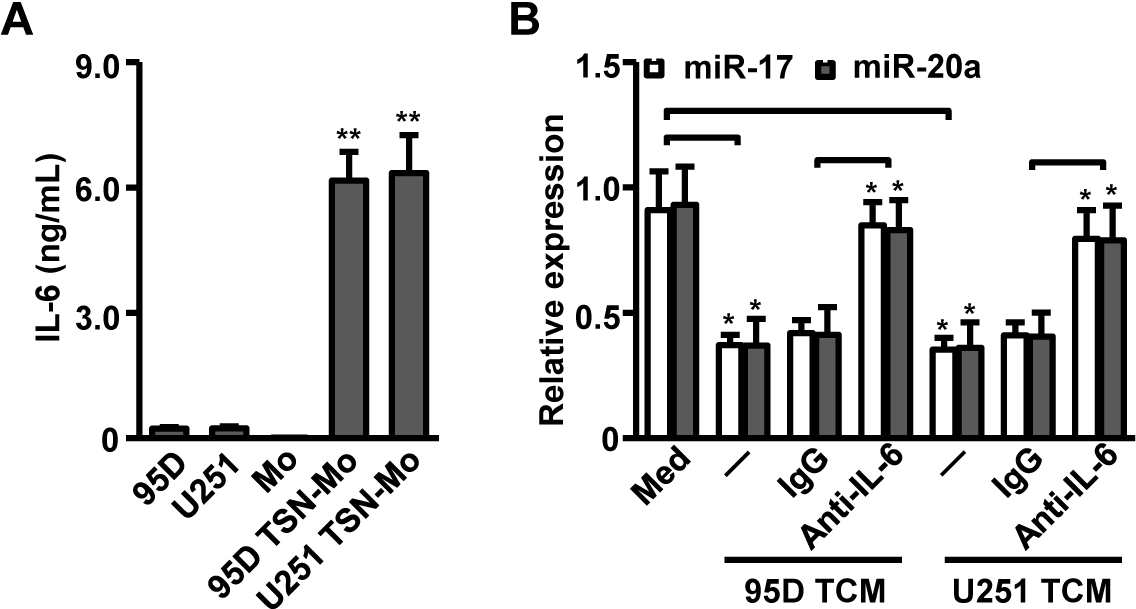

Supplement: Figure S5 — Role of IL-6 in downregulation of miR17 and miR20a in TAMs. (A) Healthy PBMC derived monocytes were treated with medium alone (MO), 95D TSN (95D TSN-MO), or U251 TSN (U251 TSN-MO) for 24 hours. IL-6 concentrations in the culture supernatants were determined by ELISA. (B) Healthy PBMC-derived monocytes were treated with medium, conditioned medium from 95D or U251 TSN-exposed macrophages (TCM), 95D or U251 TCM with anti-IL-6, or 95D or U251 TCM with IgG control for 7 days. MiR-17 and miR-20a in these cells were analyzed by qPCR. Values represent the mean ± SEM of four separate experiments. *P<0.05, **P<0.01 compared with the indicated groups. (TIF) [file pone.0077890.s005.tif]

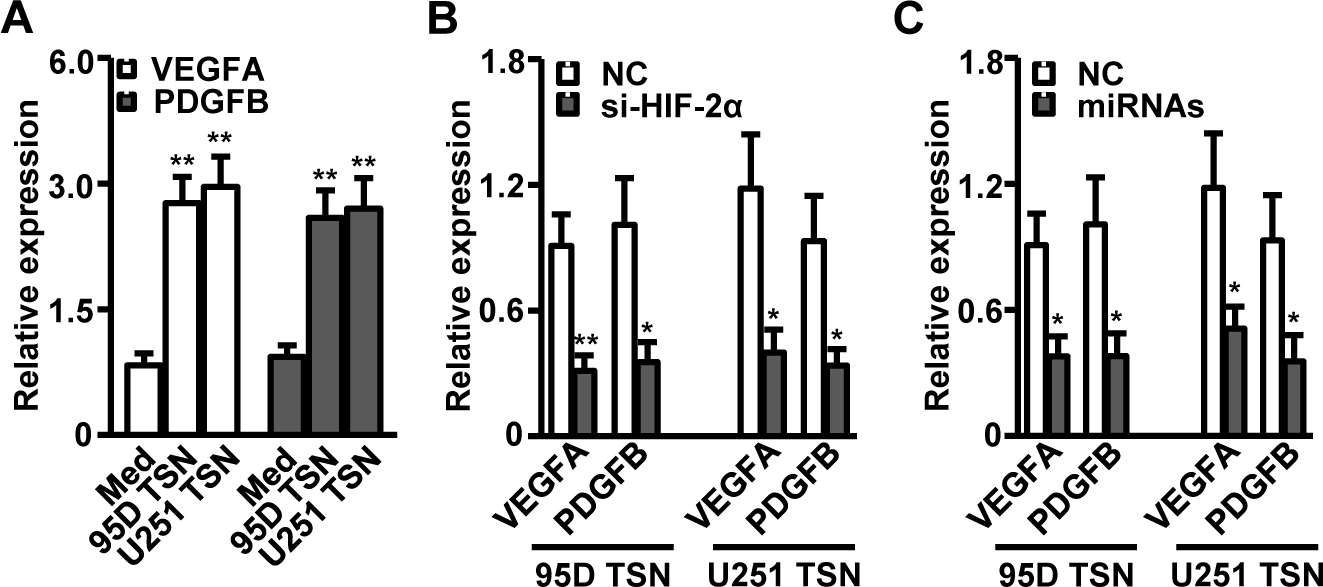

Supplement: Figure S6 — Regulation of VEGFA, PDGFB expression by HIF-2α, miR17 and miR20a in macrophages treated with 95D or U251 TSN. (A) Healthy PBMC derived monocytes were treated with medium alone, 95D TSN, or U251 TSN for 7 days. (B, C) Healthy PBMC derived monocytes were treated with 95D or U251 TSN and transfected with NC, si-HIF-2α (B), or miR-17 and miR-20a mixture (miRNAs) (C). Levels of VEGFA and PDGFB mRNA expression in these cells were analyzed by qPCR. Values represent the mean ± SEM of four separate experiments. *P<0.05, **P<0.01 compared with the indicated groups. (TIF) [file pone.0077890.s006.tif]
