# Supplementary material for: MicroRNA-17, 20a Regulates the Proangiogenic Function of Tumor-Associated Macrophages via Targeting Hypoxia-Inducible Factor 2α
Source: PLoS One. 2013 Oct 23;8(10):e77890. doi: 10.1371/journal.pone.0077890 (PMC3806827; doi:10.1371/journal.pone.0077890)
Supplement: Table S1 — Clinical characteristics of the 26 HCC patients. (DOCX) [file pone.0077890.s007.docx]

**Table S1. Clinical characteristics of the 26 HCC patients**

| **Patient characteristics** | **Value** |
| --- | --- |
| No. of patients | 26 |
| Age (years): median, range | 42, 28-70 |
| Gender: male/female | 23/3 |
| HBsAg: negative/positive | 5/21 |
| Cirrhosis: absent/present | 6/20 |
| AFP (ng/ml): ≤ 25/> 25 | 11/15 |
| Tumor multiplicity: solitary/multiple | 23/3 |
| Tumor size (cm): ≤5/＞5 | 4/22 |
| Vascular invasion: absent/present | 24/2 |
| TNM stage: I-II/III-IV | 15/11 |

Abbreviations: HBsAg, hepatitis B surface antigen; AFP, α-fetoprotein.
