# Supplementary material for: MicroRNA-17, 20a Regulates the Proangiogenic Function of Tumor-Associated Macrophages via Targeting Hypoxia-Inducible Factor 2α
Source: PLoS One. 2013 Oct 23;8(10):e77890. doi: 10.1371/journal.pone.0077890 (PMC3806827; doi:10.1371/journal.pone.0077890)
Supplement: Table S2 — Clinical characteristics of the 5 lung cancer patients. (DOCX) [file pone.0077890.s008.docx]

**Table S2. Clinical characteristics of the 5 lung cancer patients**

| **Patient characteristics** | **Value** |
| --- | --- |
| No. of patients | 5 |
| Age (years): median, range | 57, 38-70 |
| Gender: male/female | 4/1 |
| Stage: IIB-IIIA/IIIB | 2/3 |
| Pathology: adenocarcinoma/squamous cell carcinoma | 3/2 |
| Smoking status: yes/no | 4/1 |
