# Supplementary material for: MicroRNA-17, 20a Regulates the Proangiogenic Function of Tumor-Associated Macrophages via Targeting Hypoxia-Inducible Factor 2α
Source: PLoS One. 2013 Oct 23;8(10):e77890. doi: 10.1371/journal.pone.0077890 (PMC3806827; doi:10.1371/journal.pone.0077890)
Supplement: Table S3 — Clinical characteristics of the 4 glioblastoma patients. (DOCX) [file pone.0077890.s009.docx]

**Table S3. Clinical characteristics of the 4 glioblastoma patients**

| **Patient characteristics** | **Value** |
| --- | --- |
| No. of patients | 4 |
| Age (years): median, range | 34, 24-51 |
| Gender: male/female | 3/1 |
| Preoperative KPS score: ≥80/<80 | 1/3 |
| Side of tumor: left/right | 2/2 |
| Tumor location: Frontal lobe/ Parietal lobe | 3/1 |
