# Supplementary material for: MicroRNA-17, 20a Regulates the Proangiogenic Function of Tumor-Associated Macrophages via Targeting Hypoxia-Inducible Factor 2α
Source: PLoS One. 2013 Oct 23;8(10):e77890. doi: 10.1371/journal.pone.0077890 (PMC3806827; doi:10.1371/journal.pone.0077890)
Supplement: Table S4 — Sequences of RNA and DNA Oligonucleotides. (DOCX) [file pone.0077890.s010.docx]

| **Table S4. Sequences of RNA and DNA Oligonucleotides** | | |
| --- | --- | --- |
| **Name** | **Sense Strand/Sense Primer (5'-3')** | **Antisense Strand/Antisense Primer (5'-3')** |
| **miRNA and siRNA Duplexes** | | |
| miR-17 | CAAAGUGCUUACAGUGCAGGUAG | ACCUGCACUGUAAGCACUUUAUU |
| miR-20a | UAAAGUGCUUAUAGUGCAGGUAG | ACCUGCACUAUAAGCACUUUCUU |
| si-HIF-2α | ACUGCUAUCAAAGAUGCUGdTdT | CAGCAUCUUUGAUAGCAGUdTdT |
| si-IL-6R | UUAUGAAACACCAAAGUGGdCdA | CCACUUUGGUGUUUCAUAAdTdT |
| NC | UUCUCCGAACGUGUCACGUTT | ACGUGACACGUUCGGAGAATT |
|  |  |  |
| **miRNA Inhibitors** | | |
| anti-miR-17 | CUACCUGCACUGUAAGCACUUUG |  |
| anti-miR-20a | CUACCUGCACUAUAAGCACUUUA |  |
| anti-miR-C | GUGGAUAUUGUUGCCAUCA |  |
|  |  |  |
| **Primers for 3'UTR Cloning** | | |
| HIF-2α 3’UTR-1 | GCTGAATTCTCATTTGAGTCCTACCTGCTG | GCTTCTAGAGCTTGGAAAGCAAAATCATT |
| HIF-2α 3’UTR-2 | GCTGAATTCCACAGCGTGGCTTTTCCTAA | GCTTCTAGAGTGCCATCAGACCCTCTTGG |
| HIF-2α 3’UTR-3 | GCTGAATTCCTGGACTGAGGAGGAGGCTG | GCTTCTAGATTCAAGTTCTGCCACCCTGT |
|  |  |  |
| **Primers for RT-PCR** | | |
| HIF-2α | TTGGAGGGTTTCATTGCC | AGAAGTCCCGCTCTGTGG |
| VEGFA | TCCTGGAGCGTGTACGTTG | CACGTCTGCGGATCTTGTAC |
| PDGFB | CTCGATCCGCTCCTTTGATG | CGTTGGTGCGGTCTATGAGG |
| ACTB | CGCGAGAAGATGACCCAGAT | GGGCATACCCCTCGTAGATG |
